# QuickStats

**Published:** 2015-08-21

**Authors:** FROM THE NATIONAL CENTER FOR HEALTH STATISTICS

**Figure:**
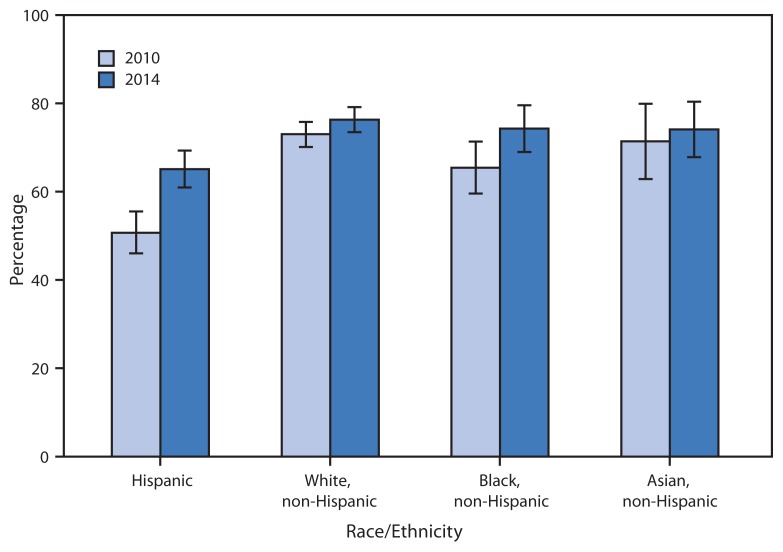
Percentage of Adults Aged 19–25 Years with a Usual Place of Care,* by Race/Ethnicity^†^ — National Health Interview Survey, United States, 2010 and 2014^§¶^ *Based on a question in the Sample Adult section that asked, “Is there a place that you usually go to when you are sick or need advice about your health?” Adults who indicated that the emergency department was their usual place for care were considered not to have a usual place of health care. ^†^ Categories shown are for non-Hispanic respondents who selected one racial group; respondents had the option to select more than one racial group. Hispanic origin refers to persons who are of Hispanic ethnicity and might be of any race or combination of races. Only selected groups shown in graph. ^§^ Estimates are based on household interviews of a sample of the civilian, noninstitutionalized U.S. population and are derived from the Sample Adult component. ^¶^ Percentages shown with 95% confidence intervals.

From 2010 to 2014, the percentage of persons aged 19–25 years who had a usual place to go for medical care increased for Hispanics (50.7% to 65.1%) and non-Hispanic blacks (65.4% to 74.3%). In 2010, among persons aged 19–25 years, non-Hispanic blacks (65.4%) were less likely than non-Hispanic whites (73.0%) to have a usual place to go for medical care; however, in 2014, no significant difference between the two groups was found. In 2010 and 2014, Hispanic adults aged 19–25 years were the least likely to have a usual place to go for medical care.

**Source:** National Health Interview Survey, 2010 and 2014 data. Available at http://www.cdc.gov/nchs/nhis.htm.

**Reported by:** Michael E. Martinez, MPH, MHSA, bmd7@cdc.gov, 301-458-4758; Brian W. Ward, PhD; Patricia F. Adams.

